# Beyond fission and fusion—Diving into the mysteries of mitochondrial shape

**DOI:** 10.1371/journal.pbio.3002671

**Published:** 2024-07-01

**Authors:** Noga Preminger, Maya Schuldiner

**Affiliations:** Department of Molecular Genetics, Weizmann Institute of Science, Rehovot, Israel

## Abstract

Mitochondrial shape and network formation have been primarily associated with the well-established processes of fission and fusion. However, recent research has unveiled an intricate and multifaceted landscape of mitochondrial morphology that extends far beyond the conventional fission–fusion paradigm. These less-explored dimensions harbor numerous unresolved mysteries. This review navigates through diverse processes influencing mitochondrial shape and network formation, highlighting the intriguing complexities and gaps in our understanding of mitochondrial architecture. The exploration encompasses various scales, from biophysical principles governing membrane dynamics to molecular machineries shaping mitochondria, presenting a roadmap for future research in this evolving field.

## Abstract 2

Mitochondria come in many shapes and sizes. This Unsolved Mystery navigates the diverse processes that influence mitochondrial shape and network formation, highlighting the intriguing complexities and gaps in our understanding of mitochondrial architecture.

## Introduction

**Fig 1:**
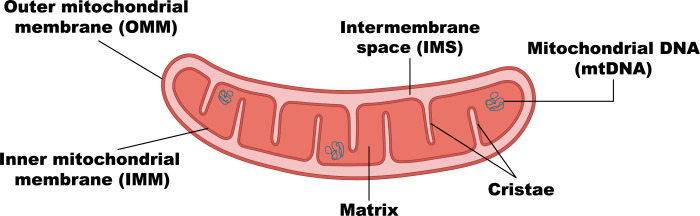
Glossary of mitochondrial components. An illustration depicting the major components of mitochondria. The OMM forms the outer boundary of the mitochondrion, and the IMM lies within. Cristae, invaginations of the IMM, serve as the primary site for ATP production. The OMM and IMM are separated by a gap named the IMS. The IMM encloses the matrix, the central compartment where numerous metabolic processes occur. mtDNA resides within the matrix, encoding essential proteins for mitochondrial function. IMM, inner mitochondrial membrane; IMS, intermembrane space; mtDNA, mitochondrial DNA; OMM, outer mitochondrial membrane.

Using a simple light microscope, already over a century ago, it was demonstrated that the mitochondrion (**Fig 1**) is a shape-shifting organelle [1]. However, it was not until the development of genetically encoded fluorophores (such as the green fluorescent protein (GFP)) and the advancement of live-cell microscopy that the dynamic nature of mitochondria and their capacity to form an interconnected, reticular architecture—referred to as the mitochondrial network [2]—truly came into focus. These technological advancements allowed researchers to track mitochondrial dynamics in real time, unveiling intricate processes such as fission (where 1 mitochondrion divides into 2) and fusion (where 2 mitochondria connect to give rise to a single mitochondrion) [3]. Hence, it became evident that mitochondria are continuously adapting their individual shape, the complexity of their network and their positioning within cells in response to various cellular and environmental cues.

Being able to visualize fission and fusion as they occur, as well as the mitochondrial network shape, opened the way for biochemical and genetic investigation of the molecular machinery governing these processes. The molecular era of this field started with the identification of the first fusion protein, Fzo, in *Drosophila melanogaster* [4], followed by the characterization of homologs in yeast [5,6] and in mammals [7]. The key mediators of fission, Dnm1 in yeast and DRP1 in mammals, were then characterized as well [8,9], and since then, thousands of papers have been published on the discovery of additional core factors and regulators of these processes. As these have been intensively reviewed (see [10–14]), we will not discuss them here.

Moreover, as new factors important for mitochondrial fission and fusion were discovered, it became clear that preserving the network structure plays a pivotal role in essential processes like repairing mitochondrial DNA [15], facilitating the interchange of mitochondrial material [16,17], and enabling the turnover of damaged mitochondria [18]. It was therefore of no surprise when fission and fusion were shown to have fundamental impact on the cellular and organismal levels and when mutations in these factors were demonstrated to have severe physiological implications [16,19,20]. Overall, the last few decades have been focused on creating a deep understanding of the fission and fusion machinery from basic cellular mechanisms all the way to pathophysiology.

An additional breakthrough in our understanding of mitochondrial shape arose from the discovery of the mitochondrial contact site and cristae organizing system (MICOS), a multiprotein complex required for cristae formation and maintenance, as well as for the connection between the outer mitochondrial membrane (OMM) and the inner mitochondrial membrane (IMM) [21–23] (**Fig 1**). The characterization of another pivotal contributor to cristae structure, the F_1_F_0_-ATP synthase, organizing into ribbon-like rows of dimers along cristae ridges and inducing high membrane curvature [24–26], contributed to the mechanistic understanding of the processes enabling the unique shape of mitochondrial internal membranes. The GTPase, optic atrophy 1 (OPA1), protein was also found to modulate the shape of mitochondria by catalyzing IMM fusion and contributing to cristae biogenesis and maintenance [27–30]. Beyond shaping by proteins, it has also been demonstrated that the IMM lipidome plays a pivotal role in shaping cristae. One of the predominant lipids involved in sustaining cristae structure is the mitochondria-specific lipid, cardiolipin. Cardiolipin is a conical-shaped lipid that was suggested to localize to highly curved membrane regions and promote the intrinsic curvature of the IMM [31]. Defects in IMM shaping proteins and lipids have been demonstrated to exert profound effects not only on cristae architecture but also on the overall morphology of the organelle [32–35].

Understanding the diverse shapes and dynamic networks of mitochondria holds profound significance in elucidating the complexities of cellular function. Mitochondria play an essential role in numerous cellular processes, including ATP production by energy conversion, calcium (Ca^2+^) homeostasis, apoptosis regulation, and more. The varied morphologies exhibited by mitochondria not only reflect their functional adaptability and responsiveness to cellular demands but also suggest that specific shapes might confer functional advantages to mitochondria themselves under various conditions. Disruptions in mitochondrial shape and dynamics have been implicated in stress and pathological conditions, such as neurodegenerative disorders, cancer, and metabolic diseases [36–39]. Therefore, unraveling the mechanisms governing mitochondrial shape provides invaluable insights into the broader landscape of cellular physiology and pathology, offering potential avenues for treatment and therapeutic innovation.

Although fission, fusion, and cristae formation contribute dramatically to the shape of mitochondria, it is important to recognize that there are additional factors that play a part in mitochondrial morphology and dynamics that have been less studied, or even overlooked. These include those affecting the various shapes that a single mitochondrion can adopt, the factors regulating and executing the varying extent of branching and connectivity in the network, and factors governing external shaping, such as pulling and tethering forces by contact sites with other organelles and cytoskeletal elements. As there are many open questions yet to be answered in this field, here, we will highlight and discuss the gaps in our current knowledge, as well as outline future research directions that could lead to a more profound comprehension of mitochondrial shape and dynamics, beyond fission and fusion.

## The shape of an individual mitochondrion

**Fig 2:**
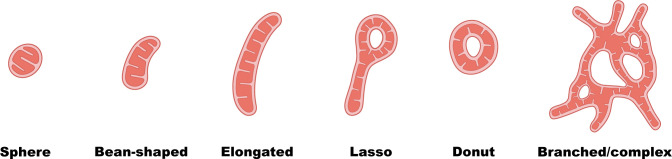
The spectrum of mitochondrial shapes. Schematic representations showcasing the diversity of mitochondrial shapes ranging from simple spheres or bean-shaped rods to circular mitochondria and intricate networks.

The basic mitochondrial unit is typically considered to be the “bean-like” linear tubular mitochondrion that appears in electron micrographs or textbooks. In fact, an individual mitochondrion can manifest a diverse range of shapes in both healthy and pathological states (**Fig 2**).

Mitochondrial morphology exhibits striking variations across different organisms, various cell types, during different metabolic conditions or circadian times and even within a single cell [40,41]. Mitochondria in hepatic tissue, for instance, exhibit structural diversity influenced by spatial positioning within the hepatic lobule—ranging from short spherical mitochondria to elongated tubules [42]. In skeletal muscle, mitochondrial subpopulations display differences in morphology, with small round mitochondria close to the sarcolemma and long tubular mitochondria located at the core of myofibers [43].

To characterize mitochondrial shape, it is important to consider a range of parameters including width, length, circularity, continuity, cristae architecture, and more. While cristae ultrastructure has earned significant attention in past studies, showing remarkable diversity across cell types and states [44], our focus here will be on exploring mitochondrial morphology on the whole-organelle level.

### Width and length: Tubule versus sphere

A single mitochondrion can exhibit a broad spectrum of shapes, ranging from a spherical form to an elongated tubular structure. How are these tubular shapes formed and sustained? Is there a functional meaning to these structural diversities? Currently, the molecular or biophysical mechanisms governing these varying mitochondrial shapes and their regulatory processes remain elusive, therefore restricting our capacity to control them and study their functional importance.

On the most spherical end of the shape spectrum is an extreme instance of a bloated sphere often referred to as “mitochondrial swelling”. This state of mitochondria, which is typically linked to mitochondrial dysfunction, can arise from the prolonged opening of the nonselective channel named the mitochondrial permeability transition pore (PTP) or alterations in the activity of ion channels such as the mitochondrial ATP-sensitive potassium channel. Ions and water are redistributed across the membranes, which expands matrix volume. The volume increase can have implications on cristae ultrastructure and lipid organization within the IMM [45,46]. PTP-induced mitochondrial swelling was shown to stimulate the cleavage of OPA1, which contributes to the change in cristae organization and mitochondrial morphology [47].

Since swelling has long become a “hallmark” for pathological states [48,49], it has potentially overshadowed additional, functional, causes for mitochondria to change shape to a more spherical structure. Spherical mitochondria can also be observed in healthy states within specific cell types, such as hepatocytes [50], or within morphologically diverse populations of mitochondria within a single cell [51]. Under physiological conditions, mild increase in matrix volume contributes to the regulation of mitochondrial metabolism and function [52,53]. Hence, the assumption that an observed spherical structure is a result of swelling, or associating any swelling as being pathological based solely on appearance, may be wrong. Rather, we suggest that functional variations should be considered and emphasize the importance of correlating structural changes with loss of membrane potential or overall altered function.

Conversely, exploring the tubular side of mitochondrial shapes begs the question of whether this occurs similarly to other established processes that shape tubules in cells, such as the shaping of the tubular network of the endoplasmic reticulum (ER). ER tubules are shaped by reticulons, REEPs, and atlastins, evolutionary conserved proteins that stabilize high membrane curvature [54–57]. A noteworthy comparison can be seen between the ER protein atlastin and the mitochondrial mitofusin (MFN) 1—both GTPases have a role in fusion (ER or mitochondrial, respectively) and share a similar structural organization with a heptad repeat domain that has been suggested to allow them to induce membrane destabilization and curvature [58–60]. Notably, mitochondrial diameter exceeds that of an ER tubule (approximately 200 to 1,000 nm compared to approximately 50 to 100 nm, respectively [61,62]); hence, proteins for stabilizing the curvature may not be required. If needed, these proteins should be able to sense larger-scale curvature (similar to septins, for example, which can sense micrometer-scale curvature [63]), or otherwise, there could be other proteins or lipids influencing the overall degree of tubularity or defining the width. Regardless of the mechanism, determining the curvature of mitochondria may be even more complex compared to other organelles since the inner and outer membrane diameter must be coordinated.

A potential external factor influencing the degree of tubularity could be mitochondrial interaction with the cell’s cytoskeletal elements or other organelles. A similar mechanism can be observed in peroxisomal elongation, where pulling forces deform the peroxisomal membrane [64]. This aspect will be addressed in more detail in subsequent sections.

However, due to the bacterial origin of mitochondria, it could very well be that intrinsic factors are the strongest modulators of shape. For example, bacterial cells grow into rounded, rod-like, or vibrioid shapes using intrinsic shape determining proteins. Bacteria utilize proteins that form cytoskeletal elements corresponding to eukaryotic tubulin, actin, and intermediate filaments [65]. Interestingly, it has been suggested that the crista junction (CJ) forming MICOS complex, which is arranged as a spiral on the IMM, may function as a “mitoskeleton” similarly to the spiral arrangements of bacterial proteins that drive tubular shaping of the cell [66]. Several controversial observations reported the presence of a subpopulation of β-actin and myosin within mitochondria, suggested to be involved in nucleoid movement [67,68] and electron transport chain complex IV function [69]. Despite the ongoing debate regarding the validity of actin localization within mitochondria, the potential contribution of intramitochondrial cytoskeletal elements to shaping remains an intriguing open question for further exploration.

Arguing that indeed intrinsic elements govern mitochondrial shaping are 2 observations. First, when mitochondria undergo a shape change from tubules to spheres triggered by elevation in cytosolic Ca^2+^ levels, which is sensed by the OMM protein MIRO1 (more on MIRO1 below), this shape transition is independent of fission, cytoskeletal elements such as microtubules and kinesin motor proteins, or PTP opening [70]. Another intriguing observation is that isolated mitochondria can retain their tubular shape when iso-osmolarity is maintained [71]. Both findings highlight possible shaping capabilities internal to mitochondria, either by a proteinaceous skeletal element or even the mere organization of the IMM into cristae.

### Circularity and linearity: Donuts, lassos, and spheroids

While an individual mitochondrion is often envisioned as a linear structure described along a single axis, the proportion of length to width alone may not capture the full complexity of certain mitochondrial shapes. Rather, it is important to also consider the circularity of mitochondria—here used to define the presence of continuous loops or closed forms. These unique morphologies include “donuts” (ring-like mitochondria) and “lassos” (ring structures with tails) (**Fig 2**). These unconventional shapes are most observed during oxidative stress (after CCCP administration [72]) or exposure to cold temperatures [73], but they also occur frequently in healthy, nonstressed cells [74].

Previous research on donut- or lasso-shaped mitochondria has described their formation due to self-fusion events within a linear mitochondrion. Fusion of tip-to-tip (TTT) would create donuts and fusion of tip-to-middle (TTM) would create lassos (more on types of fusion below). However, it has also been suggested that some of the ring structures observed in 2D microscopy images are actually indented spheroids with no through hole, formed independently of fission and fusion [72].

Numerous questions persist about these structures. The precise nature of their formation remains ambiguous—whether they result from an active and regulated process of shape change or whether they emerge as byproducts of dysregulation in mitochondrial dynamics. Additionally, their functional significance in both physiological and pathological conditions is yet to be determined. Circular shapes may offer a functional advantage, potentially decreasing diffusion distance and facilitating an increased diffusion-based flow of particles within the matrix. Whether this circularity serves as a mechanism enabling mitochondria to respond to diverse stress conditions, such as temperature maintenance under cold stress or the induction of mitochondrial stress responses during oxidative stress, remains unknown and warrants further comprehensive investigation.

### From form to function: Physiological implications of the individual mitochondrial shape

A wide range of mitochondrial shapes and transitions between them are observed under various physiological states and conditions. However, the functional significance of each shape is still not fully understood.

One clear example where shape plays a functional role is the process of mitophagy, the selective removal of damaged mitochondria, crucial for mitochondrial and cellular health [75]. During mitophagy, the autophagosome, a globular organelle with a diameter of approximately 1 μm [76], engulfs mitochondria. Hence, targeted mitochondria are required to be of small globular shape to fit. Indeed, it has been proposed that mitochondria undergo fragmentation and become small and spherical before mitophagy [77,78]. Changes in shape are also coupled to mitochondrial motility, since mitochondria need to be in a suitable shape and size for easy transportation [79].

An additional important aspect accompanying mitochondrial shape changes is the alteration in the ratio between outer membrane surface-area to total volume (SA_OMM_/V). While IMM surface area can be increased through cristae folding, providing a larger ATP-generating surface to meet energetic demands [80,81], alterations in the overall organelle shape can result in varying ratios of OMM surface area to volume. When the volume is held constant, distinct mitochondrial shapes will exhibit distinct SA_OMM_/V values. Spherical compact shapes possess the smallest SA_OMM_/V, which increases as the mitochondrion transitions into a more narrow, elongated morphology. SA_OMM_/V values might hold functional significance, potentially serving as a key parameter for size and shape control, similarly to cell size control in bacteria [82]. Different SA_OMM_/V values essentially indicate a different OMM perimeter relative to the capacity to respire and may be required under varying conditions, depending on factors such as the need for interaction with cellular surroundings or for a biochemical and signaling platform (requiring high surface area, hence high SA_OMM_/V), the requirement for proliferation of internal structures (thus requiring low SA_OMM_/V) and the availability of building blocks to increase either surface area (membrane lipids and proteins) or volume (matrix material and proteins).

Indeed, shape transitions are observed upon changing conditions, such as introduction of oxidative stress [83] or during developmental processes [84]. Considering the interplay between SA_OMM_/V and observed changes in mitochondrial shape across different states and conditions, along with the metabolic demands that arise with them, could enhance our understanding of the functional benefits and the physiological relevance of each mitochondrial shape.

## From individual to network

**Fig 3:**
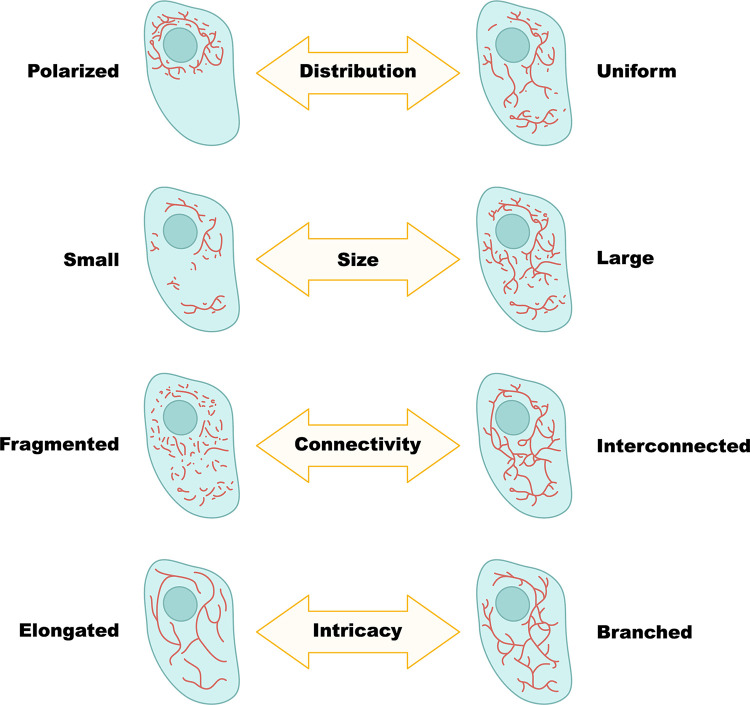
Variability in mitochondrial network architecture. Mitochondrial networks exhibit a spectrum of architectures, characterized along multiple axes (from top to bottom): cellular distribution, ranging from uniform distribution or polarization at specific cell regions; network size, reflective of total mitochondrial mass in the cell; connectivity within the network, spanning from isolated mitochondria to one interconnected network; and network intricacy, describing the amount of branching points or junctions, resulting in network morphology that varies from elongated to highly branched structures.

Although mitochondria can exist as discrete individual organelles of various shapes, they often come together to form a branched and interconnected network. The term “network” is sometimes used to describe the entire set of mitochondria in the cell, but we will use it here specifically to refer to the tubular reticulum structure of connected mitochondria. The connectivity of mitochondria, or the network extent, can vary between cells and even within the same cell under different conditions, ranging from isolated individual mitochondria to a highly connected mesh [85]. This structural variation can be seen correlated with metabolic state, for example, as seen when comparing respiring and nonrespiring cells [2]. Network structure has immense physiological implications such as for inheritance, for mitophagy, and for maintaining mitochondrial DNA fidelity [86,87]. The mitochondrial network can vary in additional topological aspects, such as the number of branching points, the location or distribution in the cell, and the degree of dynamics (**Fig 3**), and for these parameters, the impact on activity and physiology is less clear. Network dynamics relies on the rate of topology changes and the speed of forming or removing connections. To gain a comprehensive understanding of mitochondrial network formation, it is crucial to examine the intricate interplay of processes that influence it.

### Not just more of the same: Tip-to-tip versus tip-to-middle fusion

Mitochondrial fusion is a key driver of network formation, and it can be categorized into 2 distinct subtypes: TTT fusion, where 2 mitochondria fuse at their ends, resulting in an elongated mitochondrion within a network or a donut shape following self-fusion events; and TTM fusion, where one end of a mitochondrion fuses with either the side of another, forming a 3-way junction or with its own side, creating a lasso-shaped mitochondrion. It was demonstrated in U2OS cells and human primary fibroblasts that most fusion events (75%) are TTM fusions [88,89]. Interestingly, actin seems to be associated more with TTM fusion events [89]. Furthermore, ABHD16A, an ER protein regulating fission and fusion nodes at ER–mitochondria contact sites, affects the 2 fusion subtypes differently; its deletion reduces TTM fusion but has a lesser impact on TTT events [88]. These findings underscore the mechanistic differences between the 2 fusion subtypes, highlighting their distinct regulation (and potentially even machinery) and clarifying that much more research is required to understand how these 2 types of fusion are distinguished and regulated.

**Fig 4:**
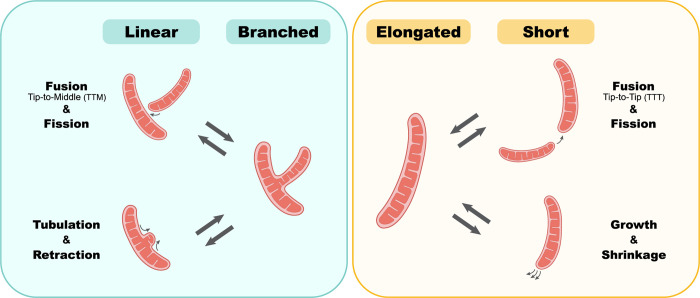
Diverse processes affecting mitochondrial morphology. The formation of branched and elongated mitochondrial shapes can be achieved through distinct cellular processes. While fission and fusion have been intensely studied in these contexts, branched morphology can be achieved not only by TTM fusion but also by active tubulation (left). Similarly, elongated mitochondrial shapes can be formed not only by TTT fusion events but also by growth and extension of an existing mitochondrion (right). Similarly, retraction or shrinkage can counterbalance these processes to restore the original shape.

The balance between TTT and TTM fusion has significant implications for network structure. A prevalence of TTT fusions leads to an elongated morphology, while favoring TTM fusions results in a branched interconnected network with numerous 3-way junctions in a mesh like manner (**Fig 4**). A possible example to a case where one fusion subtype will be favorable may be in cell types with a characteristic mitochondrial morphology, such as the long tubular mitochondria found in dendrites [90]. Currently, our understanding of the specific mechanisms, timing, and regulation for each fusion subtype is limited. It remains unclear whether this balance is tightly regulated or arises from stochastic behavior. Thus, it is crucial to distinguish between fusion subtypes and gain a deeper understanding of the molecular machineries, conditions, timing, and regulatory factors governing each type.

### Attach or branch? Fusion/fission versus tubulation/retraction

Fusion and fission processes influence both the shape and size of individual mitochondria, as well as the overall structure and connectivity of the mitochondrial network [91]. However, a junction can be formed without the need for fusion at all. An alternate way is for mitochondria to undergo tubulation, forming new branches that extend from the main body of the mitochondrion [92] in a fusion-independent manner. Similarly, removal of 3-way junctions can also occur in a fission independent manner by simple retraction (**Fig 4**).

**Fig 5:**
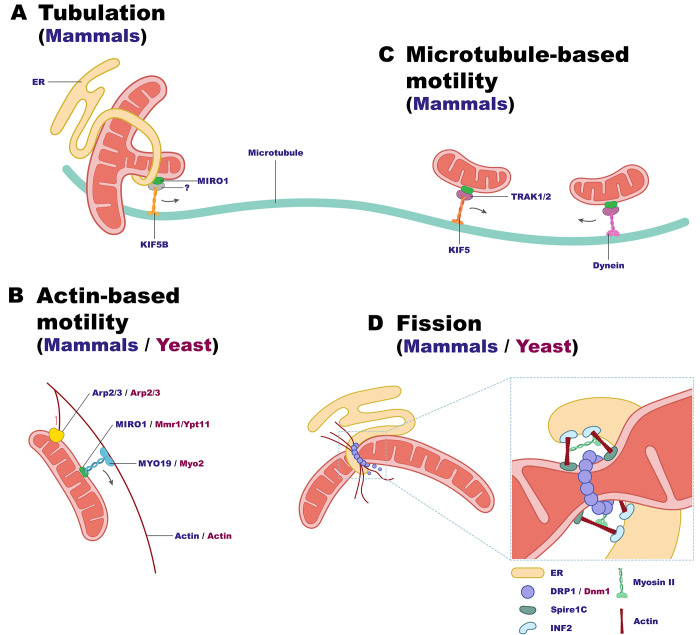
Cytoskeleton–mitochondria interactions. Cytoskeletal elements are involved in several essential mitochondrial processes. Known mechanisms and proteins in mammals (blue) or yeast (purple) are indicated by their respective color. (**A**) Mitochondrial tubulation in mammals is carried out by the kinesin KIF5B binding the mitochondrial receptor MIRO1 at ER–mitochondria contact sites. This pulling along microtubules results in the extraction of thin tubules out of mitochondria, allowing the formation of a new branch and a 3-way junction. An adaptor protein has not been shown in this specific process, hence is labeled with “?”. (**B**) Actin-based movement involves myosin motors. MYO19 in mammals is recruited and stabilized by MIRO proteins. In yeast, the Myo2 motor binds either Mmr1 or Ypt11, allowing transportation of mitochondria from the mother cell to the bud. The mitochondria-associated Arp2/3 complex supports polymerization of actin (red arrow indicates the direction of actin flow). (**C**) Mitochondrial motility along microtubules in mammals is carried out by kinesins (anterograde, towards the plus-end of microtubules) and dynein (retrograde transport, towards the minus-end). Kinesin-1 family proteins (KIF5) and dynein motors interact with the adaptors TRAK1/TRAK2 and MIRO1. (**D**) Mitochondrial fission is initiated at ER–mitochondria contact sites, where actin is polymerized by INF2, with Spire1C suggested to stimulate nucleation. DRP1/Dnm1 is recruited to the site to form a constriction ring. Myosin II participates in preconstriction and DRP1 recruitment, although its localization is unclear and is depicted here in simplicity.

To date, very little data exists about tubulation and retraction. One demonstrated mechanism was shown to occur at the cell periphery of mammalian cells and is carried out by the kinesin KIF5B and the mitochondrial GTPase MIRO1 that acts as a KIF5B receptor on mitochondria at ER–mitochondria contact sites [92,93] (**Fig 5A**). The specific function at the cell periphery suggests the existence of additional tubulation machineries yet to be discovered. MIRO1 also participates in mitochondrial trafficking by binding motor proteins from the KIF5 family [94–96]. Its interaction with kinesins is regulated by Ca^2+^, facilitated by its Ca^2+^-sensing EF hand domains, thus linking cytoplasmic Ca^2+^ sensing to both mitochondrial transport and tubulation (further elaboration on MIRO1-mediated motility below).

Although both fusion and tubulation can lead to the same final mitochondrial structure, they clearly must engage distinct molecular machineries and regulatory factors. Notably, despite the external structural similarities of the products of these reactions, there is a significant internal difference between the mitochondria formed by these 2 processes. Fusion allows for content mixing between 2 distinct mitochondria while keeping the total mass of mitochondria in the cell unaffected. In contrast, tubulation involves *de novo* growth of mitochondria and alteration of total mitochondrial mass. The choice between fusion and tubulation is, therefore, complex, requiring coordination at various levels, including structural considerations, recruitment of specific machinery, and broader cellular regulatory mechanisms to ensure resource availability required for *de novo* growth. While fission and fusion are currently the primary focus in the study of network shaping and dynamics, it is essential to start considering also tubulation and retraction. Hence, understanding the interplay between fusion/fission and tubulation/retraction, the timing of each, and the mechanisms governing their selection remains a key area of research.

### Maintaining equilibrium: Balancing shape and mass changes during tubulation and retraction

Tubulating mitochondria undergo rapid and continuous extension and retraction. To do so without damaging the lipid packing of the OMM, tubulation and retraction need to either maintain overall surface area (for example, by reducing the extent of the tips, thereby negating the need for new lipids) or be coupled with synthesis or import of new lipids essential for surface area growth. If no new lipids are introduced, a tubulation event will necessitate a coordinated shortening of the original mitochondrion, allowing lipids to flow into the newly formed tubule during extension, and vice versa during retraction.

Conversely, in cases where the size of the original mitochondrion remains unaltered, tubulation demands a rapid lipid flux to support the increase of lipids required for extension and the decrease during retraction of tubules. Lipid flux often occurs at membrane contact sites, involving lipid transfer with other organelles or the IMM [97]. A significant portion of mitochondrial lipid trafficking occurs at ER–mitochondria contact sites. In yeast, the ER–mitochondria encounter structure (ERMES), a heterotetrameric complex with lipid transport modules, tethers the ER and the OMM and contributes to the lipid transport between them [98]. Proteins from the VPS13 family, conserved from yeast to humans, act as tunnels for mass lipid transport (distinguishing it from shuttle-like lipid transporters that typically transfer one lipid at a time) at various contact sites, including the human ER–mitochondria contact [99]. Notably, mitochondrial tubulation events are predominantly found at ER–mitochondria contact sites [93]. This proximity might allow the rapid and high-flux lipid flow needed to support membrane expansion and retraction during tubulation events. Importantly, lipid flux is not enough as growth and shrinkage of overall mitochondrial mass through the process of tubulation or retraction also necessitate an adaptive response from proteinaceous components of the membranes as well as the matrix, which must adjust in size accordingly. Moreover, they would necessitate IMM and cristae reshaping. How such adjustments are coordinated is yet completely understudied.

An intriguing possible mechanism to assist in reducing mitochondrial mass during retraction events, including both lipids and proteins, could lie in the formation of mitochondrial-derived vesicles (MDVs) or mitochondrial-derived compartments (MDCs), which can rapidly remove OMM mass or both OMM and IMM in the case of MDVs [100,101]. However, the role of MDVs or MDCs in mitochondrial reshaping has not yet been established.

## No network is an island

The mitochondrial network is shaped by many signals and processes that are intrinsic to the organelle itself (such as fission, fusion, tubulation, and retraction). However, mitochondria are not isolated entities but are intricately embedded in the cellular landscape, forming physical and regulatory interactions with all other components. These interactions impact and influence the various characteristics of the network (**Fig 3**) from the outside. Hence, it is essential to explore the role of cellular elements external to mitochondria in shaping the network and defining its architecture.

### Shaping through contacts with other organelles

Mitochondria form membrane contact sites with all other organelles [102]. Many contacts have already been well characterized from yeast to humans, such as the ER–mitochondria contact [98], the vacuole/lysosome–mitochondria contact [103], and the peroxisome–mitochondria contact [104]. Contact sites are essential for mitochondrial function but also for normal mitochondrial morphology. For instance, the mitochondria–ER–cortex anchor (MECA), which tethers mitochondria, the ER, and the plasma membrane (PM) in yeast [105] has been shown to be essential for correct mitochondrial network morphology. Disrupting MECA leads to a collapsed network morphology with high motility due to the loss of attachment to the cortex [106–108]. In addition to physical anchoring, it has been suggested that tethering mitochondria to the PM generates the membrane tension required for Dnm1 to perform fission [109].

Another critical contact for mitochondrial shape is that between ER and mitochondria. This contact regulates lipid transfer, Ca^2+^ homeostasis, reactive oxygen species signaling, and more [110]. In addition, it was shown to play a crucial role in maintaining the mitochondrial network shape, as its loss causes a total collapse of the mitochondrial network and defects in its cellular distribution [98]. The ER–mitochondria contact is also where fission and fusion machinery converge to form hotspots for membrane dynamics [111]. In mammalian cells, the fusion protein MFN2, together with MFN1, not only facilitates mitochondrial fusion but also plays a pivotal role in establishing and maintaining contact sites between the 2 organelles [112–114]. This dual functionality suggests a coordinated regulation allowing the spatial and molecular integration of fusion events and the formation of contact sites. Additionally, as discussed above, dynamic tubulation of mitochondria occurs at these contacts [93]. It was recently suggested that 2 distinct types of fission exist, each governed by contacts with different organelles—ER-mediated fission occurs at the midzone of the mitochondrion and leads to mitochondrial proliferation, while peripheral fission is preceded by lysosomal contact and facilitates the degradation of mitochondria through mitophagy [115].

Given the wide range of functions of each contact, it is often difficult to clearly define whether their impact on mitochondrial shape is direct (for example, due to them serving as physical anchors or scaffolds, exerting mechanical forces to maintain and stabilize the structure of the network, affecting fission and fusion or enabling lipid fluxes) or indirect (for example, due to their metabolic contribution to mitochondrial processes). The last years have mapped many of the tethers for these contacts providing the tools to start answering these questions using genetic tools.

### Shaping by cytoskeletal elements

The cytoskeleton plays a significant role in various aspects of mitochondrial dynamics and shaping. In yeast, the actin cytoskeleton serves as a scaffold for anchoring mitochondria, directing their movement inside the cell, and ensuring proper inheritance during cell division [116,117]. The driving forces for movement are generated by Arp2/3 complex-stimulated actin polymerization [118] (**Fig 5B**). Conversely, in mammalian cells, long-range mitochondrial movement is mainly facilitated by molecular motors (kinesins and dyneins), which actively move mitochondria along microtubules [119] (**Fig 5C**). These motor proteins associate with mitochondria through the OMM proteins MIRO1/MIRO2 and their motor adaptors TRAK1/TRAK2. During cytokinesis, the centromeric protein CENPF is recruited to mitochondria by MIRO1/2 and associates with microtubules, promoting mitochondrial movement and proper cellular distribution into daughter cells [120].

In mammalian cells, shorter-range movements are also mediated by the actin cytoskeleton and myosin motors [121], particularly the mitochondria associated motor MYO19, which is recruited and stabilized by MIRO proteins [122] (**Fig 5B**). In addition to its role in mitochondrial motility, actin also plays a crucial role in mediating mitochondrial fission. Actin polymerizes at ER–mitochondria contact sites, controlled by the ER-bound Inverted formin-2 (INF2) [123], leading to preconstriction of the OMM and to the recruitment of DRP1 that further constricts and splits the mitochondrion [124] (**Fig 5D**).

The mechanical forces exerted on mitochondria by the cytoskeleton can influence both individual mitochondrial shape and network architecture in additional ways. One direct way is by providing pulling forces and physical tension on the membrane, actively participating in shaping processes. Additionally, the cytoskeleton may have an indirect regulatory role, since moderate mechanical stress at the cellular level has been shown to enhance mitochondrial dynamics. This includes increased expression of fusion proteins and recruitment of the fission machinery, suggesting a complex interplay between mechanical forces and mitochondrial behavior [125].

## Conclusions and future perspectives

The study of mitochondria, once confined to observations under simple light microscopes, has evolved dramatically over the past century. Using imaging at high resolutions coupled with complex image analysis along mathematical and physical simulations, we can now coherently characterize the dynamic nature of these organelles in a way that can be modeled and understood. However, in our pursuit of understanding mitochondrial dynamics, it is crucial to recognize that the field has been primarily dominated by investigations into fission and fusion. While these processes undoubtedly play a crucial role in shaping mitochondria, these organelles are far more than mere binary entities dictated by these processes alone.

The current state of the field presents numerous unanswered questions and offers potential research avenues awaiting further exploration (**Box 1**). To gain a deeper understanding, we must examine mitochondrial shape at all scales, starting from the biophysical principles that govern membrane dynamics, exploring the molecular machineries responsible for various mitochondrial shapes, and delving into the intricate whole-cell pathways maintaining and remodeling network architecture. We anticipate that this comprehensive exploration will reveal new layers of regulation and complexity that govern these fascinating organelles and will further demonstrate the profound functional importance of mitochondrial shape and network architecture in both health and disease.

**Box 1. Open questions**

**The shape of an individual mitochondrion**

- What are the cellular machineries required for shaping tubular, spherical, or circular mitochondrial structures? Are there shaping proteins outside or inside mitochondria yet to be discovered?
- What regulates the decision and the extent of shape transitions—elongation of a sphere to tubule or vice versa, formation of ring-like or spheroid shapes?
- Do different mitochondrial shapes serve specific functional roles? Under what circumstances would each shape be preferred? Are there functional costs and benefits associated with SA_OMM_/V alterations in different shapes?

**From individual to network**

- What cellular factors define whether and where to create a new branch?
- Are there distinct mechanisms for tubulation of peripheral mitochondria and perinuclear mitochondria? If so, what are they? And what is the benefit of having 2 distinct machineries?
- What regulates and creates the balance between TTT to TTM fusion? When does each type of fusion occur?
- What controls the balance between fusion and tubulation as a means to create branching? How are changes in lipid mass and matrix volume coordinated with shape changes during tubulation and retraction?
- Is there a functional significance to having junctions in mitochondria? How do elongated and meshed networks differ functionally?

**No network is an island**

- What are the mechanical roles for membrane contact sites in the shaping and stabilization of the network, and are there distinct mechanical functions for different contact sites?
- What is the interplay between mechanical forces exerted by the cytoskeleton and their impact on mitochondrial shape and network architecture?
- Which factors coordinate the role of contact sites and the cytoskeleton in the mechanical shaping of the mitochondrial network?
- What are all the indirect ways by which cytoskeletal shape and function influence mitochondrial shape and network formation?

## Abbreviations

CJ: crista junction
ER: endoplasmic reticulum
ERMES: ER–mitochondria encounter structure
GFP: green fluorescent protein
IMM: inner mitochondrial membrane
INF2: Inverted formin-2
MDC: mitochondrial-derived compartment
MDV: mitochondrial-derived vesicle
MECA: mitochondria–ER–cortex anchor
MFN: mitofusin
MICOS: mitochondrial contact site and cristae organizing system
OMM: outer mitochondrial membrane
OPA1: optic atrophy 1
PM: plasma membrane
PTP: permeability transition pore
SA_OMM_/V: outer membrane surface-area to total volume
TTM: tip-to-middle
TTT: tip-to-tip
